# Egoistic versus Prosocial Decision Making: an Eye Movement Data Report

**DOI:** 10.1038/s41597-024-04083-5

**Published:** 2025-01-22

**Authors:** Anastasia Peshkovskaya

**Affiliations:** 1 Laboratory of Experimental Psychology, Tomsk State University, Tomsk, Russia; 2 Mental Health Research Institute, Tomsk National Research Medical Center, Russian Academy of Sciences, Tomsk, Russia

**Keywords:** Human behaviour, Social behaviour

## Abstract

Eye tracking data are highly promising in revealing novel and valuable evidence on human behavior and decision making. Data descripted in this article were collected in fourteen experiments with SMI eye tracking glasses in individual and social decision making conditions. The dataset is available on Harvard Dataverse and include data of 14 subjects with 4,180 visual behavior metrics summary and 3,744 eye moment records in decision-related areas of attention. Data may be applicable in computational models of oculomotor activity to explain decision process and predict its outcomes.

## Background & Summary

Vision is a synergistic effect of eye sensing and brain processing mechanisms. Extensive fundamental research considered a tight coupling between visual perception and cognition^1,2^. Studies linked visual perception with higher cognitive functions such as visual spatial attention and memory^3,4^. Decades of eye tracking research revealed that eye movements and decision making are tightly related^5,6^. Eye movement metrics has been useful in providing evidence of mechanisms that drive reward-driven attention, information search and choice^7,8^. Investigations on eye movements enable a fine-grained analysis of the behavior reflecting an escalatory decision value^9,10^, a decision in preference formation^11–13^, and multi-alternative decision making^14^.

Some farther evidence suggested a great potential of neuroscience-based metrics in investigating decision making in social environments^15,16^. Together with a functional perspective, studies employed electroencephalography^17^, fMRI^18^, neurochemical^19^, and, more recently, eye tracking measures to understand a social modulating role on decisions and social contexts of decision making^16^. Eye movement data considered to be highly promising in revealing novel and valuable evidence on social influence on attention, behavior, and choices^20–22^.

Considering these two insightful research directions in Visual and Decision Neuroscience, I present data of visual behavior and its dynamics in experimental investigation for decision making in individual and social conditions.

## Methods

Data collection was performed in fourteen experiments with the SMI eye tracking glasses (by SensoMotoric Instruments GmbH), a mobile eye tracking device with a 120 Hz binocular sampling rate capturing eye movements with 0.5° gaze tracking accuracy and 80° horizontal, 60° vertical gaze tracking range.

The sample is available on Harvard Dataverse and include data of 14 subjects (7 women) aged between 20 and 40 years (M 23.7, SD 6.2) with 4,180 visual behavior metrics and 3,744 eye moment records in decision-related areas of attention. All experiments were conducted in both individual and group conditions, and applied data filters covered individual versus group decision making conditions as well as pro-self subject behavior versus prosocial behavior. The study procedures involving human participants were approved by the Ethics Council of Tomsk State University (Approval 101–2019 on 2 September, 2019) and adhered to the tenets of the Declaration of Helsinki. The methods in the study were in accordance with relevant guidelines, and a written informed consent was obtained from all participants.

As an experimental framework, Prisoner’s Dilemma (hereinafter PD), an essential example of a social dilemma widely used in behavioral and decision making studies^21,23–26^, was employed to investigate pro-self and prosocial decision making. In PD, individuals can either cooperate or defect by making prosocial or pro-self decisions. Both would benefit from mutual cooperation but defecting or making egoistic pro-self choices would be more rewarding in terms of payoff as a PD game outcome.

In experimental procedure, twelve participants were invited to a computer classroom where they took their seats at the computer desks and were instructed and then completed the participant consent form.

Stage 1. One participant of twelve was equipped with the mobile eye tracking glasses device (hereinafter ETG). The ETG were calibrated with a standard 1-point calibration procedure to ensure the high quality of the participant’s eye movements tracking. Then, all the experiment participants proceeded to a computer-based PD game (20 trials). The participants were able to move to the next trials only after all of them had made their choices. No one knew who the opponents were, and in each round the pairs changed randomly. The current trial result and the overall game result were displayed on computer screens after each trial. Eye recording started from the beginning of the first trial of the PD game. After the game was completed, the participant with ETG took off the ETG device.

Stage 2. Participants were involved in a social interaction through the communication and group formation, when two groups of six individuals each were created by participants based on their common interests, hobbies, and etc. This laboratory model combines the classic social psychology minimal group paradigm with group manipulations that cause a sense of social attachment^27,28^.

Stage 3. Participants took their seats at the computers. They were instructed that they would be asked to play PD again, but this time their partner would be a random member from the newly formed group of six people. The participant who was equipped with ETG at the Stage 1 put on the ETG and the standard calibration procedure were held once again. Then all the participants proceeded to PD, which consisted of 22 trials. The result of each trial and the total personal result for the game were displayed on the participants’ computer screens after each trial of the game.

**Fig. 1:**
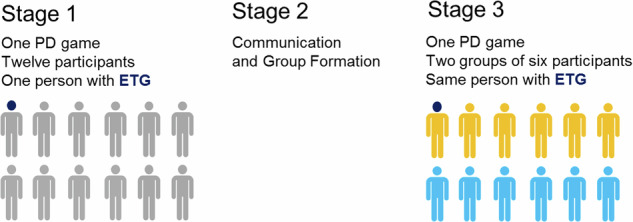
Research Design employed for generation the data reported in this article.

The research design description presented graphically with Fig. 1.

**Fig. 2:**
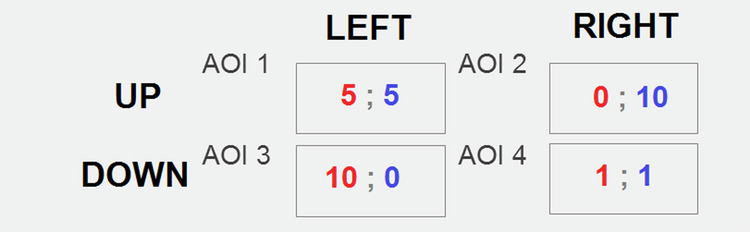
PD’s Areas of Interest employed for AOIs-based aggregating the eye movement indicators.

All the trials during Stage 1 and Stage 2 employed the same PD game with unchanged payoff matrix (presented with Fig. 2).

## Data Records

The Semantic Gaze Mapping tool was used for aggregating each participant’s eye movement data. After Semantic Gaze Mapping-based data aggregation, eye movement quantitative metrics were exported with BeGaze software with the following variables:

- Revisits – the amount of eye gaze returns to the already scanned area;
- Fixation Count – the number of eye gaze fixations in the specified area per second;
- Average Fixation Duration – the mean value of the fixation time in the specified area;
- Fixation Time – the time of eye gaze fixation in the specified area, as a percentage of the time that the respondent looked at the specified area;
- Saccade Count – the number of eye saccades in the specified area per second;
- Saccade Frequency (count/s) – the number of saccades per second;
- Saccade Latency Average (ms) – the average reaction time of a visually guided saccade;
- Dwell Time – the total time that the subject looked at the specified area, as a percentage of the time of the demonstration of the whole area;
- Scanpath Length (px) is the total length of all saccades in gaze trajectory.

In addition, cells of a payoff matrix in the PD game were employed in analysis as so-called Regions of Interest (ROIs)^29^ or Areas of Interest (AOIs)^21^ (Fig. 2). Markup was done before the experiments were conducted. Information on AOI Name, AOI Order, AOI Size (px), and AOI Coverage (%) is available in the dataset. Alike the above, aggregate measures such as fixation counts, saccades, dwell times, and others were registered on each PD’s Area of Interest and then exported.

Generated and deposited data are divided into two datasheets – eye movement records summary and AOIs-based eye movement records. Both are available in a deposited data file on Harvard Dataverse^30^ and are ready for data processing.

## Technical Validation

All data were collected in one laboratory using the unified protocols and tasks. The PD game interface as well as the PD game payoff matrix structure was the same during all experiments and experimental stages. All the participants recruited in the experiments had normal or corrected-to-normal vision. A standard 1-point eye calibration procedure and SMI software of eye movement data pre-processing were in accordance with relevant technical guidelines and ensured reliability of all the variables and technical quality of the dataset. Together, these homogeneities minimize the variation of the experimental environment, tasks, procedures, and participants.

## Usage Notes

As a description of data potential for reuse, I highlight the important data application directions. First analytical glance at eye movement data revealed that, in general, eye behaviour changed throughout the experimental stages. By the Stage 2, when participants made their decisions in PD game under social condition, their eye fixation and saccade frequencies were increased. Time delays between saccades were reduced, and the total length of gaze path became consistently shorter. Such a dynamic may be interpreted by perceptual learning effect as participants had already experienced all the visual environment of the PD game by the experimental Stage 2 and basic stimuli elements were unaltered across the PD task. However, further interpretation demands a bit deeper and comprehensive look into the data, and social versus individual decision-making conditions as well as their impact on decisions that were made should be considered.

In addition, eye movement data on visual environment of the PD game, which was set as regions of interest (particularly, PD matrix cells), deliver a unique clue on eye movement patterns during pre-decision visual information processing. For example, reported data showed that longer fixations were more common for participants with pro-self decisions than for those who made prosocial ones. While eye fixation is universally considered to be linked to a task processing model, which assumes that the eye fixates the referent of the symbol being operated on^31^, the obtained evidence can be explained by the consideration that pro-self strategy probably demands more cognitive effort. However, this consideration should be confirmed with other eye metrics of participants who showed ‘egoism’ in decision making task.

Along with that, further directions of data reuse may include sophisticated modelling of pro-self and prosocial decision making based on eye movement data. These computational models may employ neural networks and deep learning to enrich current knowledge of eye-brain decision making mechanisms.

## Data Availability

No custom code was used to generate or process the data described in the manuscript.
